# Determinants of chronic obstructive pulmonary disease severity in the late-elderly differ from those in younger patients

**DOI:** 10.1186/s13104-015-1810-8

**Published:** 2016-01-04

**Authors:** Mizuha Haraguchi, Hidetoshi Nakamura, Mamoru Sasaki, Masaki Miyazaki, Shotaro Chubachi, Saeko Takahashi, Koichiro Asano, Paul W. Jones, Tomoko Betsuyaku, the Keio COPD Comorbidity Research (K-CCR) Group

**Affiliations:** Division of Pulmonary Medicine, Department of Medicine, Keio University School of Medicine, 35 Shinanomachi, Shinjuku-ku, Tokyo, 160-8582 Japan; Department of Respiratory Medicine, Saitama Medical University, 38 Morohongo, Moroyama-machi, Iruma-gun, Saitama, 350-0495 Japan; Division of Pulmonary Medicine, Department of Medicine, Tokai University School of Medicine, 143 Shimokasuya, Isehara, Kanagawa 259-1193 Japan; Division of Clinical Science, St. George’s University of London, Cranmer Terrace, London, SW17 0RE UK

**Keywords:** Chronic obstructive pulmonary disease, Comorbidity, Elderly, Symptom, Emphysema

## Abstract

**Background:**

Although the age range of chronic obstructive pulmonary disease (COPD) patients is broad, few studies have focused on the effects of age on disease characteristics.

**Methods:**

Keio University and affiliated hospitals established an observational COPD cohort. Patients were assessed using high resolution computed tomography (CT) to quantify emphysema, health status using the COPD assessment test (CAT) and the St. George’s Respiratory Questionnaire (SGRQ), spirometry, echocardiogram, dual X-ray absorption of bone, biomarkers and comorbid diagnoses. We examined the characteristics of COPD patients aged 75 and over compared with patients below 75.

**Results:**

A total of 443 patients comprising 252 patients aged <75 years and 191 patients aged ≥75 years, were enrolled. Emphysematous changes on CT and prevalence of possible pulmonary hypertension were greater in late-elderly patients. The slope of the relationship between CT emphysema densitometry score and forced expiratory volume in 1 s was significantly less steep in the late-elderly than the younger patients (p = 0.002). CAT and total SGRQ scores and the frequency of long-term oxygen therapy were significantly higher in the late-elderly with moderate airflow obstruction compared to those of the younger in the same grade, although the opposite was seen in late-elderly patients with very severe airflow obstruction. Hypertension, aortic aneurysm, prostatic hypertrophy, anemia, and cataract are more prevalent in late-elderly patients.

**Conclusions:**

Elderly COPD patients show a varied age-related pattern of disease that warrants specific attention in clinical practice above and beyond assessment of airflow limitation.

*Trial registration* Clinical trial registered with the University Hospital Medical Information Network (UMIN000003470, April 10, 2010)

**Electronic supplementary material:**

The online version of this article (doi:10.1186/s13104-015-1810-8) contains supplementary material, which is available to authorized users.

## Background

Chronic obstructive pulmonary disease (COPD), one of the most prevalent health conditions, is the fourth leading cause of death worldwide [1]. COPD morbidity and mortality have been increasing in many countries, in part due to an aging population world-wide [2]. By the mid-21st century, the chance of living beyond 60 years will be 98 % in Japan/Oceania, 82 % in Western Europe, and 69 % in China [3]. Relative to the 2011 world population, recent United Nations projections estimate that by 2100 the number of people aged >60 years will triple, with an eightfold increase in those >80 [1]. However, although increases in life expectancy and the size of the elderly population during the past several decades might explain the current increase in COPD, the relationship may be more complex, including factors such as differential susceptibility to tobacco, anatomic and systemic differences, behavioral differences, and differences in response to available therapeutic modalities.

Japan is a super-aged society, ranked first in the world. The late elderly (aged ≥75 years) accounted for 11.6 % of Japan’s population in the 2010 national population census. In recent years pulmonary and primary care physicians in Japan have been more ready to diagnose and treat COPD patients of advanced age, and although it is known that COPD currently mostly affects middle-aged and elderly people, few studies have focused on how the features of COPD differ by age.

In the past, COPD severity was simply classified based upon the % forced expiratory volume in 1 s (FEV_1_), because it was believed that the majority of patients followed a path of disease progression in which the severity of the disease tracked the severity of the airflow limitation [4]. However, it has become clear in recent years that comprehensive assessment requires more than FEV_1_ measurement. The Global Initiative for Chronic Obstructive Lung Disease (GOLD) has proposed an assessment for COPD treatment based on the patient’s level of symptoms and future risk of exacerbation, in addition to the severity of spirometric abnormality [5].

Very elderly patients with COPD may present differently from younger ones; they may have a different pattern of comorbidities, and a different survival rate after acute exacerbations. Physicians may also have an age bias that may affect both diagnosis and treatment. The purpose of this study was thus to examine the characteristics of COPD patients aged 75 and over compared with those aged below 75.

## Methods

### Study populations

Keio University and affiliated hospitals have established an observational COPD cohort, registered with the University Hospital Medical Information Network (UMIN000003470), for investigations of the management of COPD comorbidities [6]. All patients were clinically stable without exacerbations for at least 1 month prior to study. The protocol was approved by the Ethics Committees of Keio University on July 29, 2009 (No. 20090008) and the affiliated hospitals, and written informed consent was obtained from each patient.

### Measurement of pulmonary functions

All participants underwent spirometry when in a stable condition during the baseline examination, according to ATS protocols using an electronic spirometer [7]. Predicted values were derived from the guidelines for pulmonary function tests issued by the Japanese Respiratory Society [8]. The classification of disease severity was based on GOLD spirometric grading [2], Grade I: mild (FEV_1_/forced vital capacity (FVC) <0.70, %FEV_1_ ≥80 %), Grade II: moderate (FEV_1_/FVC <0.70, %FEV_1_ 50–80 %), Grade III: severe (FEV_1_/FVC <0.70, %FEV_1_ 30–50 %) and Grade IV: very severe COPD (FEV_1_/FVC <0.70, %FEV_1_ <30 %).

### Assessment of clinical parameters

At enrollment, a full medical and smoking history and current pharmacological treatment were obtained and clinical examinations were performed. Comorbid diagnoses were established using clinical history and examination findings, supported by a review of available medical records. The Hospital Anxiety and Depression Scale (HADS) was used, with a cut-off score of 11 points each for a probable status of anxiety or depression [9]. Gastro-esophageal reflux disease (GERD) symptoms were evaluated using a self-reported Frequency Scale for the Symptoms of GERD (FSSG) questionnaire, consisting of 12 items, with a cut-off score of 8 points for GERD [10].

### Questionnaires on health-related quality of life (QOL)

Each patient’s health-related QOL was evaluated using three questionnaires. Two were disease-specific: the COPD Assessment Test (CAT) and St George’s Respiratory Questionnaire (SGRQ) [11–14]. The CAT has been validated in Japan using the same population enrolled in the present study [6]. The Medical Outcomes Study Short-Form 36-Item (SF-36) version 2 was also used to measure the patients’ general health status [15, 16], and it was reported that COPD patients have SF-36 lower scores, representing worse health-related QOL [17]. All of the questionnaires were completed by the patients themselves, at home, in the stable state.

### Evaluation of emphysema on CT scan

Quantitative High Resolution computed tomography (HRCT) analyses of emphysema were performed. Low-attenuation areas (LAAs) using a threshold level of −950 HU were determined using a Discovery CT 750HD CT system (GE Healthcare, Tokyo), adjusting the threshold on each model of CT scan using a CT scanner test object, the Multipurpose Chest Phantom N1 “Lungman” (Kyoto Kagaku, Kyoto, Japan) [18] and calculated its percentage relative to the entire lung area (LAA%) using the workstation Lexus 64^®^ (AZE Ltd., Tokyo) [19].

### Echocardiographic evaluation

Echocardiograms were obtained using two commercially available echocardiography systems (GE Vivid7/Vivid9, GE Healthcare, Horten, Norway and iE33/Sonos7500, Philips; Amsterdam, Netherlands). A 2.5-MHz transducer was used to obtain the images in the parasternal and apical views, corresponding to the standard long-axis, and two-chamber and four-chamber images, respectively. Standard two-dimensional and color Doppler data were collected. The estimated systolic pulmonary artery pressure (eSPAP) was calculated [20], and pulmonary arterial hypertension was defined by an eSPAP ≥35 mmHg [21].

### Statistical analysis

All data are expressed as mean, unless otherwise stated. Student’s *t* test was performed to compare mean values between the two groups. Comparisons of data among the four patient groups were performed using analysis of variance (ANOVA), followed by a Tukey–Kramer post hoc analysis. Comorbidities were included as a categorical variable. A χ^2^ analysis was conducted to compare the frequencies between two groups. Relationships between quantitative data were examined using Spearman tests. Analysis of covariance (ANCOVA) were conducted to examine whether the slope of the relationship between two parameters differed between the patients aged <75 and ≥75 years. P values less than 0.05 were considered significant. All data were analyzed using the JMP version 9.0.2 software for Windows.

## Results

### Demographic measures

The mean age of the 443 COPD patients was 72.6 ± 8.2 SD years (range 43–91 years), and 92 % were males. The clinical characteristics of the subgroups of late-elderly patients (aged ≥75 years) and relatively younger patients (aged <75 years) are tabulated (Table 1). There was no significant difference between the two subgroups in smoking amount, BMI, proportion of the GOLD grades, and their treatment with medication. Current smokers were more prevalent in the younger patients.

**Table 1:** Demographic data of the COPD patients (n = 443)

	Age <75 years	Age ≥75 years	*p* value
No. of subjects	252	191	
Age (mean, years)	66.9	80.0	
Sex (male/female)	231/21	175/16	n.s.
Smoking amount (mean, pack-year)	56.9	55.6	n.s.
Current smoker (%)	17.3	7.7	0.004
BMI (mean, kg/m^2^)	22.5	22.3	n.s.
VC (mean, ml)	3365	2934	<0.001
VC (mean, % predicted)	93.8	93.5	n.s.
FEV_1_ (mean, ml)	1717	1465	<0.001
FEV_1_ (mean, % predicted)	60.5	62.1	n.s.
GOLD grade (I/II/III/IV)	52/114/67/19	41/88/48/14	n.s.
LTOT (%)	10.7	15.9	n.s.
CAT (mean)	12.5	12.7	n.s.
SGRQ symptom (mean)	36.5	37.9	n.s.
SGRQ activity (mean)	39.7	47.5	0.001
SGRQ impact (mean)	18.3	22.3	0.04
SGRQ total (mean)	27.4	32.2	0.02
Oral corticosteroids (%)	3.2	3.6	n.s.
Inhaled corticosteroids (%)	35.0	32.8	n.s.
Long-acting β2 agonists (%)	42.0	48.8	n.s.
Long-acting muscarinic antagonists (%)	64.0	58.3	n.s.

*BMI* body mass index, *VC* vital capacity, *FEV*_*1*_ forced expiratory volume in 1 s, *GOLD* Global Initiative for Obstructive Lung Disease, *LTOT* long-term oxygen therapy, *CAT* COPD assessment test, *SGRQ* St George’s Respiratory Questionnaire, *n.s.* not significant

Numbers, mean (SD), and median (range) of age in GOLD grades I to IV were as follows; I: n = 93, 71.6 (8.2), 73 (49–89), II: n = 202, 72.9 (8.6), 73 (43–91), III: n = 115, 72.9 (7.7), 74 (51–89), IV: n = 33, 72.3 (7.9), 74 (54–89). There was no significant difference in age among the GOLD grades.

### Vital capacity (VC) and FEV_1_ by GOLD grade

**Fig. 1:**
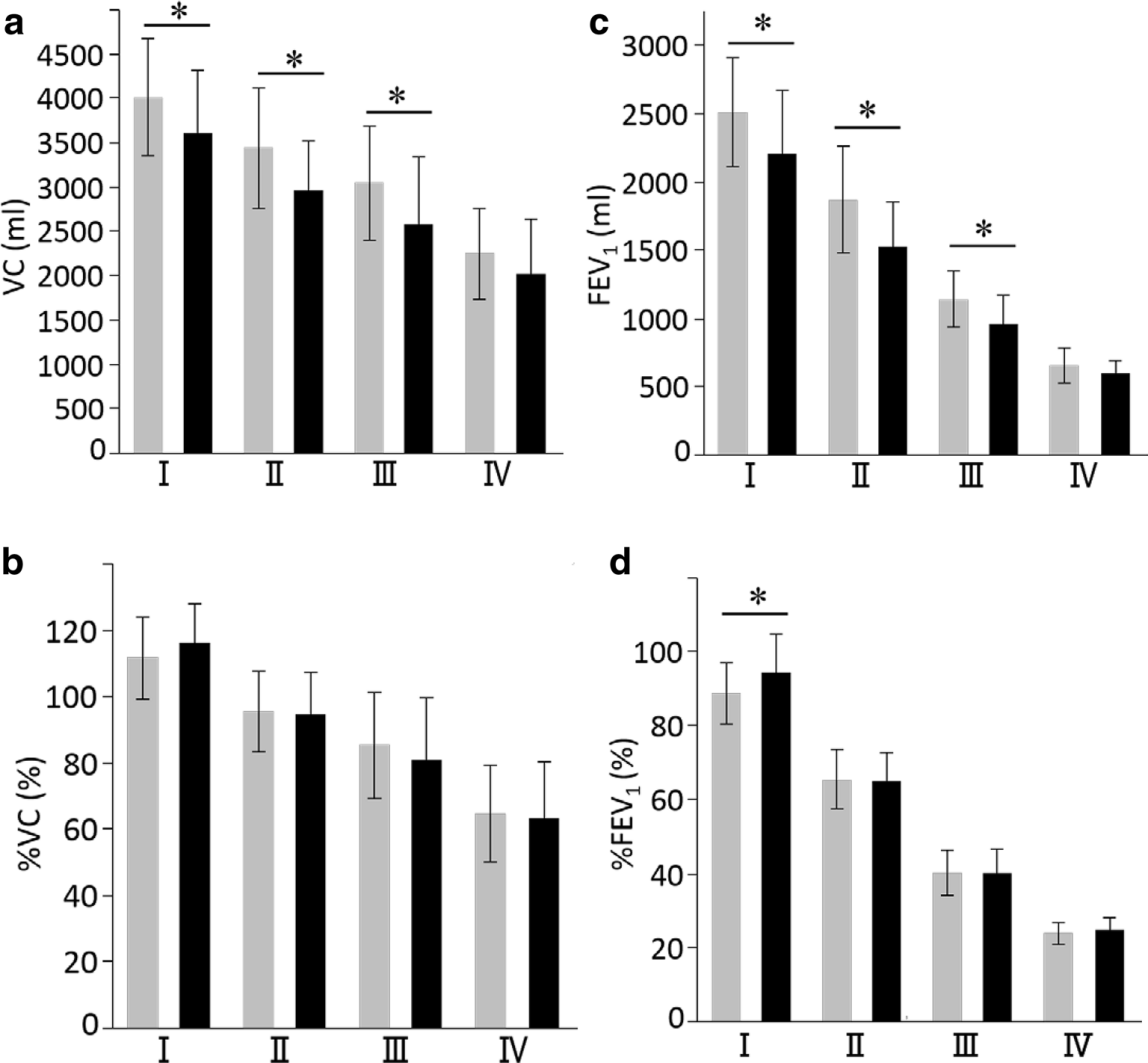
Comparisons of VC and FEV_1_ values between COPD patients aged <75 and ≥75 years with different grades of COPD. **a** VC, **b** %VC, **c** FEV_1_, **d** %FEV_1._ *Gray columns* <75 years old, *Black columns* ≥75 years old. Data are presented as mean ± standard deviation (SD). *p < 0.05

An individual’s VC and FEV_1_ are expected to fall with increasing age, premised on the repeated findings that respiratory function worsens with age. In GOLD grades I–III, the VC and FEV_1_ values were significantly lower in the late-elderly patients compared to the younger patients, but among the GOLD grade IV patients there was no difference between the two groups (Fig. 1a, c). In contrast, the mean VC % predicted and FEV_1_ % predicted were not lower in the late-elderly patients compared to the younger patients when grouped by GOLD grade (Fig. 1b, d).

### Age-related difference in the relationship between emphysema on CT scan and FEV_1_

**Fig. 2:**
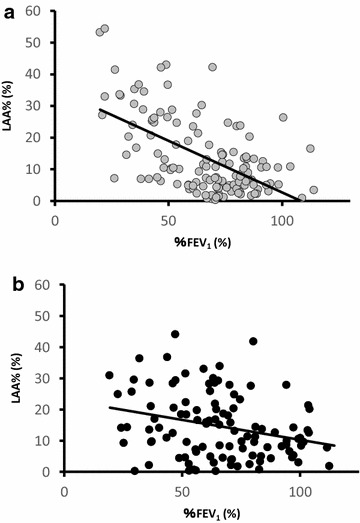
Correlation between %FEV_1_ and LAA % in each age group. **a** *Gray circles* <75 years old, **b** *black circles* ≥75 years old. *p = 0.002 comparison of the slopes between two different age groups

CT scans were performed on 246 patients enrolled at Keio University Hospital. Overall, there was a significant correlation between the degree of emphysema and %FEV_1_ (r = −0.453, p < 0.0001), but in the patients aged <75 years, the slope of the relationship between these two variables was significantly steeper than in patients aged >75 (ANCOVA, *p* = 0.002) (Fig. 2).

### Age-related difference in the prevalence of pulmonary hypertension

**Fig. 3:**
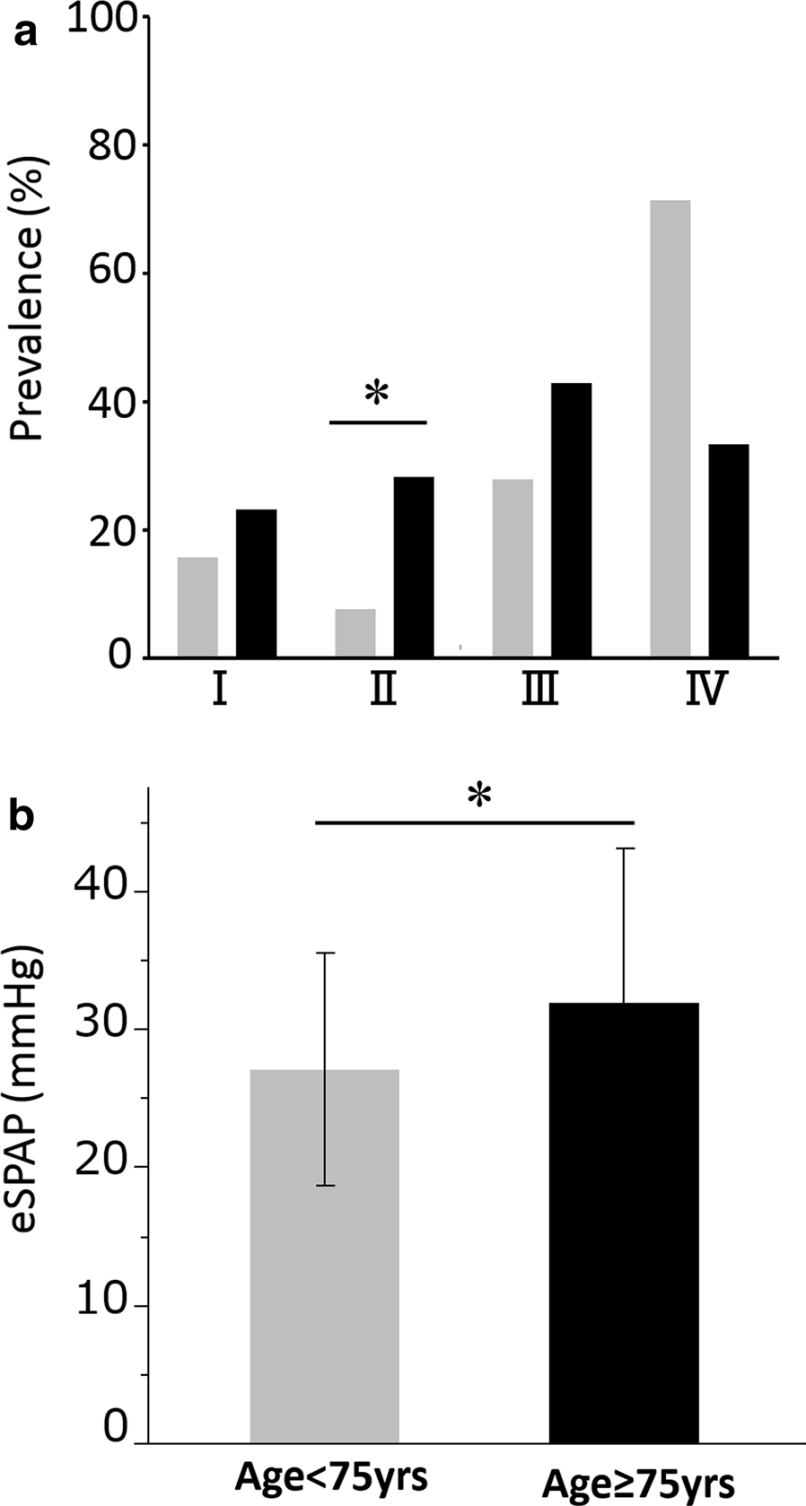
Relationships between pulmonary hypertension and age. **a** Comparisons of prevalence of possible pulmonary hypertension (eSPAP ≥35 mmHg) between COPD patients aged <75 and ≥75 years in different stages of COPD, *p = 0.02, **b** comparisons of eSPAP between COPD patients aged <75 and ≥75 years after controlling for differences in LAA %. Data are presented as mean ± SD. *p = 0.001

Echocardiography was performed on 265 patients enrolled at Keio University Hospital, and the eSPAP was measurable for 179 of these patients (72.8 %). Among the GOLD grade II patients, the prevalence of possible pulmonary hypertension was significantly higher in the late-elderly patients compared to the younger patients. (28.2 vs. 7.5 %, *p* = 0.02) (Fig. 3a). After controlling for differences in LAA % between the two groups of patients, patients ≥ 75 years had a higher level of eSPAP (31.9 vs. 27.1 mmHg, *p* = 0.001) (Fig. 3b).

### Age-related difference in health-related QOL

**Fig. 4:**
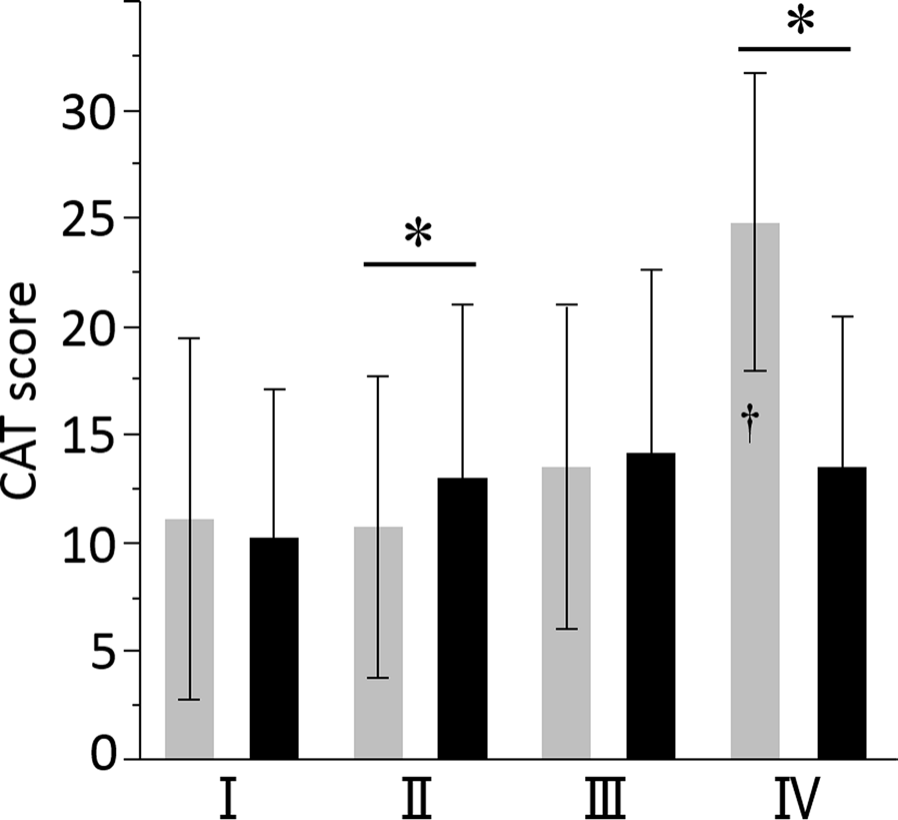
Total CAT scores of COPD patients aged <75 and ≥75 years with different GOLD grades of COPD. *Gray columns* <75 years old, *black columns* ≥75 years old. Data are presented as mean ± SD. *p < 0.05 between two different age groups. p < 0.0001 comparison between four GOLD grade groups of <75 years old, and ^†^p < 0.05 by post hoc analysis between Grade IV vs. I, II, and III groups

**Fig. 5:**
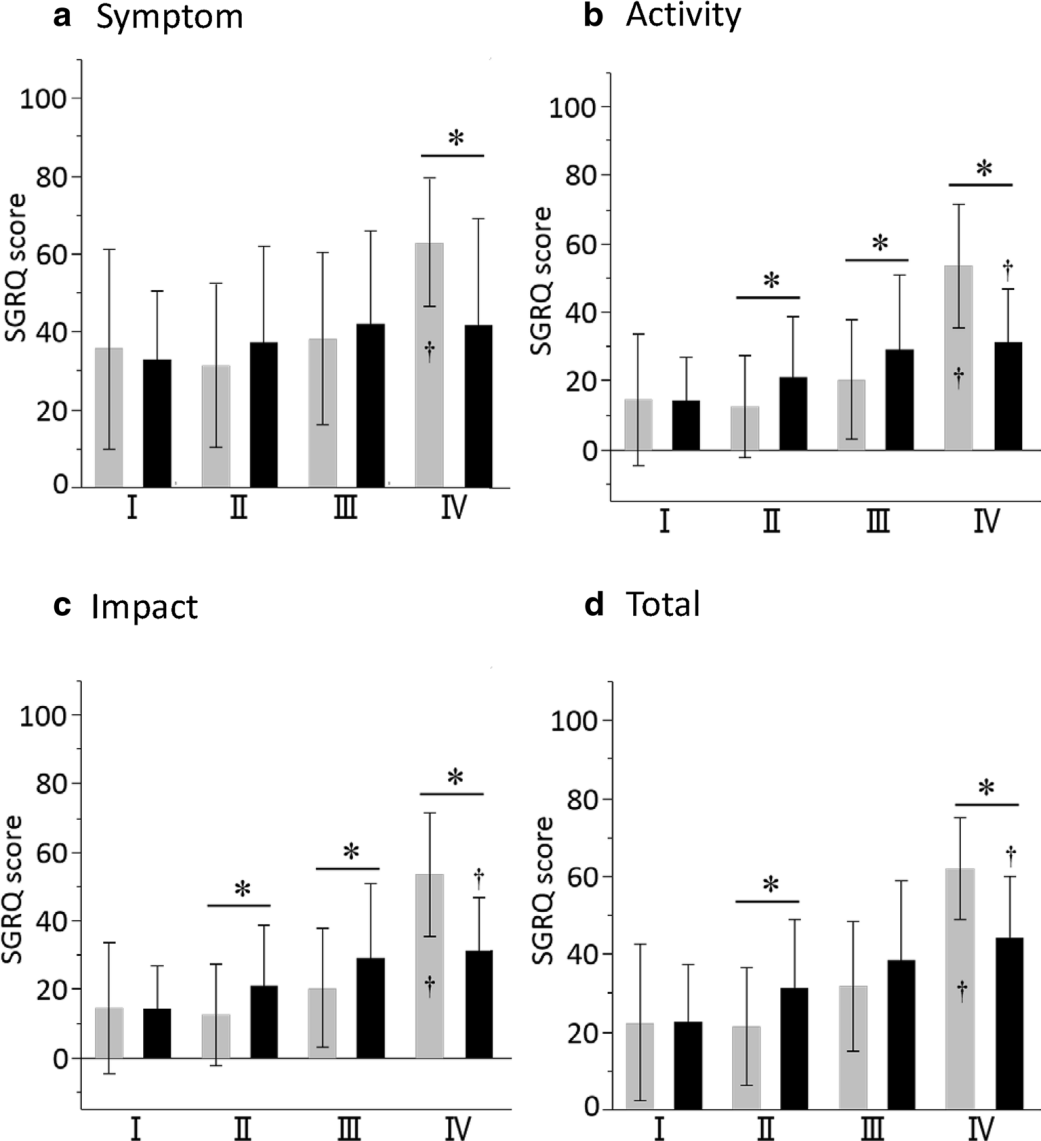
Comparisons of SGRQ scores between COPD patients aged <75 and ≥75 years in different grades of COPD. **a** Symptom scores, **b** activity scores, **c** impact scores, **d** total scores. Data are presented as mean ± SD. *p < 0.05. p < 0.001 comparison between four GOLD grade groups of <75 years old, and ^†^p < 0.05 by post hoc analysis between Grade IV vs. I, II, and III groups (**a**–**d**). p < 0.001 comparison between four GOLD grade groups of ≥75 years old, and †p < 0.05 by post hoc analysis between Grade IV vs. I and II groups (**b**), Grade IV vs. I group (**c**, **d**)

The CAT score increased (worsened) with worsening GOLD grade in the younger COPD patients (*p* < 0.0001); this was largely due to the very high score in the younger patients in GOLD Grade IV. There was no overall significant difference in CAT score between GOLD grades in the late-elderly COPD patients (*p* = 0.15) (Fig. 4). Differences between elderly and younger patients were inconsistent across the GOLD grades. The same pattern seen with the CAT were also seen in with SGRQ scores (compare Fig. 4 with Fig. 5), and with the generic questionnaire, the SF-36 (Additional file 1: Figure S1, Additional file 2: Figure S2).

### Age-related variability in the introduction of long-term oxygen therapy (LTOT)

There was no significant difference in the ratio of patients receiving LTOT between the younger and late-elderly COPD patients as a whole (Table 1), although there was a pattern of more LTOT use in the late-elderly patients in GOLD Grades I-III, but significantly less in GOLD IV patients (Table 2), perhaps because of the combined mortality impact of age, severe airflow limitation and severe hypoxia.

**Table 2:** Prevalence of LTOT among COPD patients < 75 and ≥ 75 years old

	Prevalence (%)		*p* value
	<75 years old	≥75 years old	
I	1/52 (1.9 %)	3/41 (7.3 %)	n.s.
II	4/114 (3.5 %)	13/88 (14.8 %)	0.0043
III	7/67 (10.4 %)	11/48 (22.9 %)	n.s.
IV	14/19 (73.7 %)	2/14 (14.3 %)	0.002

*n.s.* not significant

### Differences in the frequency of comorbidities by age

Some comorbidities including hypertension (46.5 vs. 29.6 %, *p* < 0.01), aortic aneurysm (6.6 vs. 1.7 %, *p* < 0.05), prostatic hypertrophy (19.7 vs. 7.7 %, *p* < 0.01), anemia (37.2 vs. 15.7 %, *p* < 0.01), and cataract (64.1 vs. 34.8 %, *p* < 0.01) were significantly more prevalent in the late-elderly compared to the younger patients (Table 3).

**Table 3:** Prevalence of comorbidities

	Prevalence (%)			*p* value
	<75 years	≥75 years	Total	
Asthma	21.1	23.0	22.0	n.s.
Interstitial pneumonia	5.4	10.9	7.8	n.s.
Lung cancer	5.2	6.0	5.5	n.s.
Other malignancies	17.6	25.7	21.2	0.05
Anxiety	8.3	5.6	7.1	n.s.
Depression	7.9	13.5	10.2	0.07
Hypertension	29.6	46.5	37.0	<0.01
Coronary artery disease	9.4	14.2	11.5	n.s.
Arrhythmia	9.9	12.0	10.8	n.s.
Chronic heart failure	4.3	8.2	6.0	n.s.
Aortic aneurysm	1.7	6.6	3.9	0.01
Diabetes mellitus	15.0	14.8	14.9	n.s.
Dyslipidemia	17.6	15.9	16.8	n.s.
Hyperuricemia	7.3	10.4	8.7	n.s.
Cerebral infarction	5.2	7.7	6.3	n.s.
Chronic renal failure	1.2	3.2	2.0	n.s.
Gastro-esophageal reflux disease	34.9	31.1	33.3	n.s.
Peptic ulcer	7.3	12.6	9.6	0.09
Chronic sinusitis	12.8	8.8	11.1	n.s.
Prostatic hypertrophy	7.7	19.7	13.0	<0.01
Liver dysfunction/Liver cirrhosis	8.7	8.8	8.7	n.s.
Collagen disease	2.1	1.7	1.9	n.s.
Anemia	15.7	37.2	25.0	<0.01
Osteoporosis	15.2	23.7	18.9	0.06
Cataract	34.8	64.1	47.9	<0.01

*n.s.* not significant

## Discussion

Japan became a super-aged society before other countries, but this trend is present in all developed and in developing countries [1]. The results of the present study suggest that COPD patients surviving to become late-elderly have a different pattern of lung function disturbance and emphysema to those who are younger. Although a strong correlation of emphysema on CT scan with spirometry was found in a large-scale cohort [22], the severity of emphysema varies widely even among patients with the same grade of COPD [19], our data suggests that age may be a factor in that variation. One of the problems in comparing different ages of COPD patients is the reliability of estimates of normal ranges for FEV_1_ in the late-elderly. VC and FEV_1_ are thought to decline linearly with age; this being the basis of equations for calculating predicted values based on age. In the present study, late-elderly COPD patients showed lower VC and FEV_1_ values compared to those in the younger patients, but they were classified in the same grade of COPD based on percentage of predicted values. These observations imply that VC and FEV_1_ were similarly reduced by age across the grades of airflow limitation in patients with COPD to those in the Japanese general population. The less steep slope of the relationship between emphysema and FEV_1_ shown in Fig. 2 may be due to a healthy survivor effect (late elderly patients with severe airflow limitation being more likely to die), but relationships between the severities of airflow limitation and emphysema can be different in the late-elderly and younger patients with COPD.

We compared health-related QOL using the CAT, SGRQ and SF-36 between the two groups at different levels of airflow limitation. Overall there was a weak but generally consistent trend towards patients with more severe airflow limitation having worse QOL. To the authors’ best knowledge, no previous studies have focused on the age-related differences of health-related QOL in patients with COPD. Age negatively affects physical function, physical role limitations and general health [23] and our study reports similar findings, although we have shown that late-elderly patients in GOLD IV have better health status than younger patients. This may reflect a healthy survival effect, since the proportion of late elderly patients GOLD IV who were on LTOT was much lower than in the younger patients with the same degree of airflow limitation. This conclusion was supported by the data on pulmonary hypertension, since for any given degree of airflow limitation, the late-elderly patients were more likely to have pulmonary hypertension, except in GOLD IV. We have previously reported that comorbid diseases such as depression, anxiety, and GERD may increase CAT scores [3]. However, there was no difference in the prevalence of such comorbidities between the late-elderly and younger patients. In contrast age-related comorbid diseases including hypertension, aortic aneurysm, prostatic hypertrophy, anemia, and cataract were not associated with CAT scores in the present study (data not shown).

One of the strengths of our cohort study is that the study population is a community-living sample. The patients enrolled provide a representative range of the socio-economic characteristics of COPD patients in Japan, registered at a general clinical practice, a university hospital or a related facility. Coincidentally, other COPD cohort studies conducted in different Japanese locality investigated participants with a similar average age [24, 25]. Indeed, COPD patients in Japan are typically elderly, and there might be a “healthy survivor effect” in their late seventies and beyond. We speculate that these older-aged COPD cohorts contain a higher proportion of people who, for whatever reasons, did not experience such serious or life-threatening comorbid problems as their younger peers. We have no reason to believe that the pattern seen in these COPD patients is a particularly Japanese phenomenon.

As COPD is more prevalent at older ages, it represents an increasing problem for public healthcare worldwide. Few studies have reported the clinical features of COPD in very elderly patients [26, 27], and the need for more research into the impact of age on this growing subpopulation of COPD patients is urgent [28]. Our study identified late-elderly COPD patients with moderate airflow limitation who manifested severe emphysema and/or pulmonary hypertension, and disabling dyspnea that were not usually complicating features of COPD patients with moderate airflow limitation for the younger patients. In part this may be because the conventional %FEV_1_ staging scheme may be misleading as a measure of severity in late-elderly patients [29]. An alternative staging strategy that accounted for age-related changes in pulmonary function and variability in spirometric performance, i.e., the lambda-mu-sigma method, demonstrated that 28.1 % of patients with severe COPD were classified as moderate using the standard GOLD grades [30].

One limitation of this study is the lack of age-matched healthy controls for comparison, although that is complicated by the fact that the elderly often live with one or more chronic conditions [31]. Another limitation is that the objective quantification of emphysema severity and pulmonary hypertension was not assessed for all of the enrolled subjects, because we had to use different types of CT scanners and echocardiography systems in each affiliated hospital. Finally, this study mainly consisted of male patients (91.6 %), and further investigations are necessary to test whether our observations are applicable to female COPD patients.

## Conclusion

The present study demonstrates that very elderly individuals present a different pattern of COPD than younger patients. This is possibly the result of different pathways of disease coupled with a healthy survivor effect. Assessments of COPD severity from a multidimensional perspective are essential for the appropriate attribution of symptoms and use of COPD-directed therapies. The results of the present study further strengthen the need for a better assessment of airflow limitation in late-elderly patients than current FEV_1_ prediction values and late-elderly COPD patients warrant specific attention in clinical practice.

## Additional files

10.1186/s13104-015-1810-8 Comparisons of SF-36 components between COPD patients aged < 75 and ≥ 75 years in different stages of COPD. Data are presented as mean ± standard deviation (SD). *p < 0.05. a: Vitality, b: Physical functioning, c: Bodily pain, d: General health perceptions.
10.1186/s13104-015-1810-8 Comparisons of SF-36 components between COPD patients aged < 75 and ≥ 75 years in different stages of COPD. Data are presented as mean ± standard deviation (SD). *p < 0.05. a: Physical role functioning, b: Emotional role functioning, c: Social role functioning, d: Mental health.

## Abbreviations

K-CCR: Keio COPD Comorbidity Research
COPD: chronic obstructive pulmonary disease
CT: computed tomography
CAT: COPD assessment test
SGRQ: St. George’s Respiratory Questionnaire
FEV_1_: forced expiratory volume in 1 s
UMIN: University Hospital Medical Information Network
GOLD: Global Initiative for Chronic Obstructive Lung Disease
FVC: forced vital capacity
HADS: Hospital Anxiety and Depression Scale
GERD: gastro-esophageal reflux disease
FSSG: frequency scale for the symptoms of GERD
QOL: quality of life
SF-36: Medical Outcomes Study Short-Form 36-Item
HRCT: high resolution computed tomography
LAAs: low-attenuation areas
eSPAP: estimated systolic pulmonary artery pressure
ANOVA: analysis of variance
ANCOVA: analysis of covariance
LTOT: long-term oxygen therapy
