# Supplementary figures and images for: Determinants of chronic obstructive pulmonary disease severity in the late-elderly differ from those in younger patients
Source: BMC Res Notes. 2016 Jan 4;9:7. doi: 10.1186/s13104-015-1810-8 (PMC4700610; doi:10.1186/s13104-015-1810-8)

## Slide 1
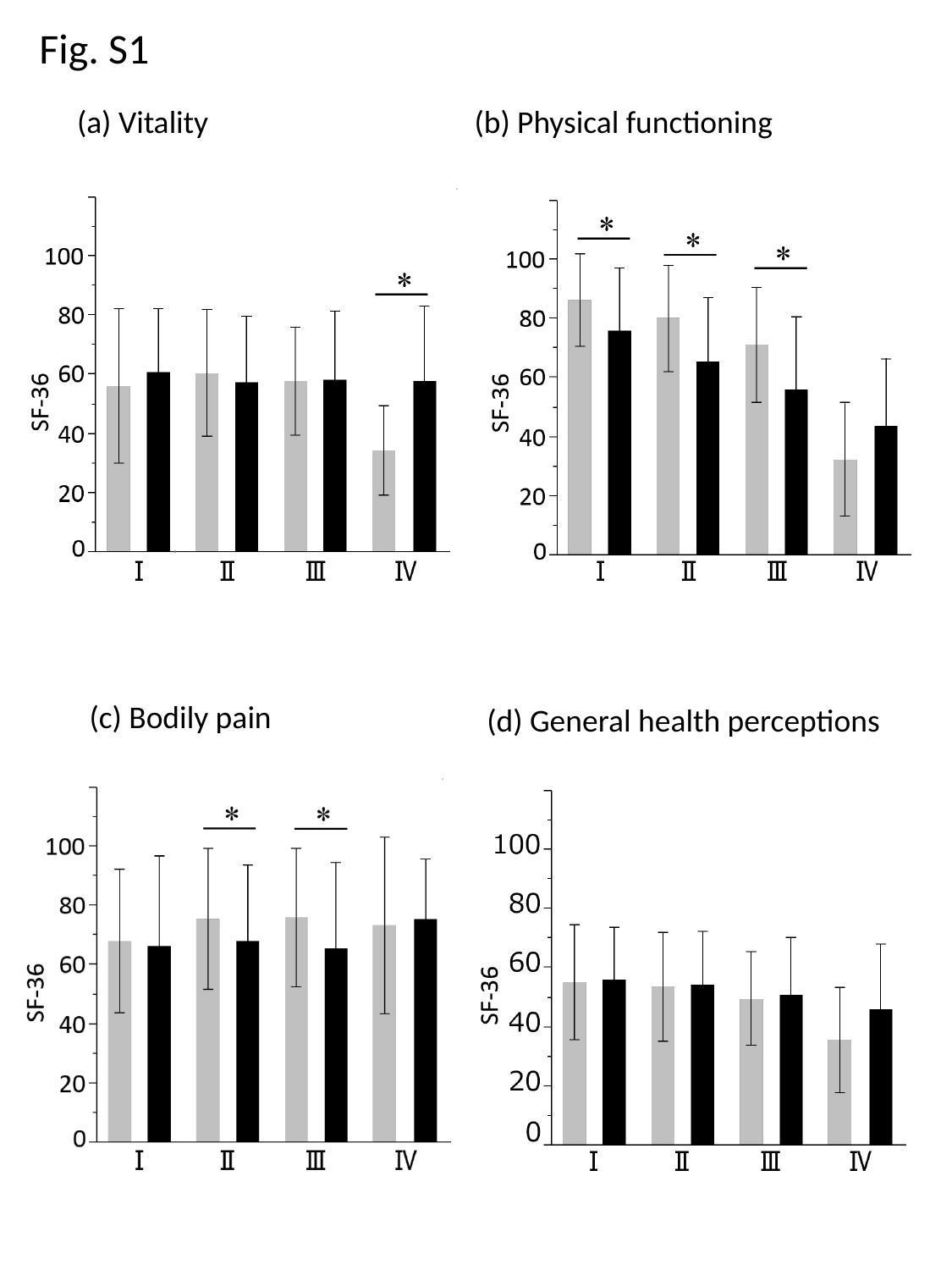

Fig. S1
(a) Vitality
(b) Physical functioning
(c) Bodily pain
(d) General health perceptions

Supplement: Supplementary file 1 — 10.1186/s13104-015-1810-8 Comparisons of SF-36 components between COPD patients aged < 75 and ≥ 75 years in different stages of COPD. Data are presented as mean ± standard deviation (SD). *p < 0.05. a: Vitality, b: Physical functioning, c: Bodily pain, d: General health perceptions. [file 13104_2015_1810_MOESM1_ESM.pptx]
