# Supplementary material for: Determinants of chronic obstructive pulmonary disease severity in the late-elderly differ from those in younger patients
Source: BMC Res Notes. 2016 Jan 4;9:7. doi: 10.1186/s13104-015-1810-8 (PMC4700610; doi:10.1186/s13104-015-1810-8)
Supplement: Supplementary file 2 — 10.1186/s13104-015-1810-8 Comparisons of SF-36 components between COPD patients aged < 75 and ≥ 75 years in different stages of COPD. Data are presented as mean ± standard deviation (SD). *p < 0.05. a: Physical role functioning, b: Emotional role functioning, c: Social role functioning, d: Mental health. [file 13104_2015_1810_MOESM2_ESM.pptx]

## Slide 1
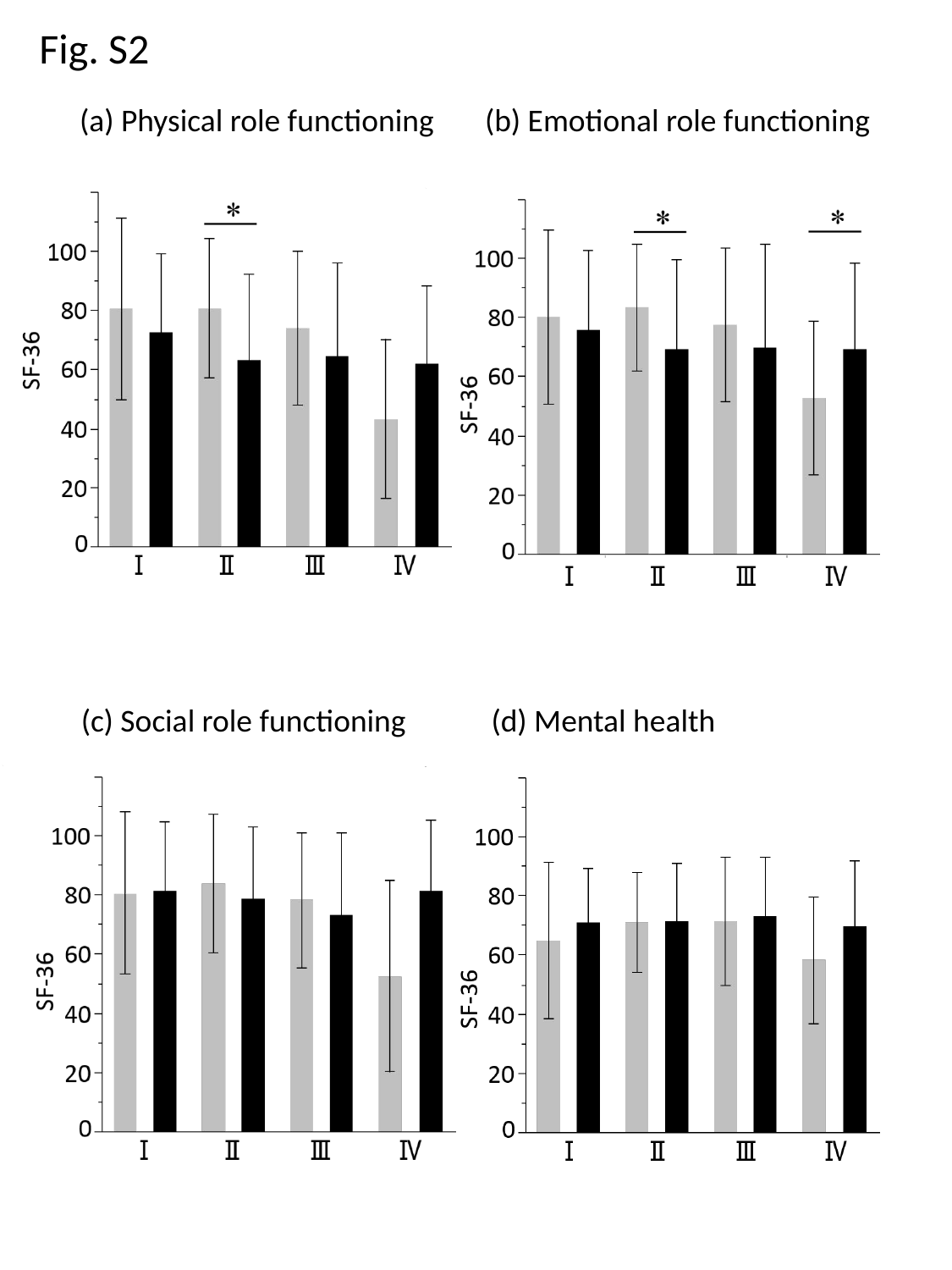

Fig. S2
(a) Physical role functioning
(b) Emotional role functioning
(c) Social role functioning
(d) Mental health
